# Cannabis and Psychosis Through the Lens of DSM-5

**DOI:** 10.3390/ijerph16214149

**Published:** 2019-10-28

**Authors:** Nathan T. Pearson, James H. Berry

**Affiliations:** Department of Behavioral Medicine and Psychiatry, West Virginia University School of Medicine, Morgantown, WV 26505, USA; jberry@hsc.wvu.edu

**Keywords:** cannabis, marijuana, psychosis, hallucinations, delusions, paranoia, schizophrenia, intoxication, DSM-5

## Abstract

Evidence for an association between cannabis and psychosis has been documented in literature in many forms including experimental studies, epidemiological data, and case series. The association has implications for psychotic outcomes ranging from mild to severe and occurring over minutes to years. Due to the huge variety of exposures and outcome measures reported, creating a coherent account of all the available information is difficult. A useful way to conceptualize these wide-ranging results is to consider the association between cannabis and psychosis as it occurs within the context of widely used DSM-5 diagnoses. In the present review we examine cannabis/psychosis associations as they pertain to Cannabis Intoxication, Cannabis-Induced Psychotic Disorder, and Schizophrenia. This allows for an understanding of the cannabis and psychosis association along something approaching a continuum. Cannabis intoxication becomes Cannabis-Induced Psychotic Disorder once certain severity and duration criteria are met and Cannabis-Induced Psychotic Disorder is heavily associated with future schizophrenia diagnoses.

## 1. Introduction

Cannabis use is common and becoming more so. There were an estimated 192.2 million users worldwide between the ages of 15–64 in 2016. This number of worldwide users represents a 16% increase compared to 2006 [1]. Legalization of cannabis for medical use has contributed to this increase [2]. In the United States, states that have passed medical cannabis laws have seen greater increases in illicit cannabis use and in cannabis use disorders compared to states that have not passed medical cannabis laws [3]. As use has increased, population-level perceptions as to the harmfulness of cannabis have decreased [4]. Tetrahydrocannabinol (THC) is of course usually considered the active ingredient but cannabidiol (CBD), several other cannabinoids, and terpenoids play a role in the pharmacology of cannabis [5]. 

Cannabis use has been associated with psychotic symptoms and disorders including schizophrenia across many populations and in many different study designs [6,7,8,9]. The nature of this association is complex and can be rife with confounders. This is especially so when looking at long-term psychotic outcomes related to cannabis use. There has been debate in the literature as to whether cannabis use is a causative factor for schizophrenia or whether the association between the two rather represents some shared vulnerability to both [8,10]. Another putative reason for the association has been that cannabis use represents an attempt by people with emerging psychosis to self-medicate their symptoms though recently that explanation has been falling out of favor as a primary explanation [7,9]. 

While this review focuses on cannabis proper we should note before moving on that synthetic cannabinoid use is a growing clinical concern due to the significant prevalence and potential for severe health effects beyond what is seen with cannabis [11,12]. A 2013 survey of 50,000 US high school students reported 6.4% of students with past-year synthetic cannabinoid use compared to 25.8% of students with past-year cannabis use [13]. The US Army Substance Abuse Program in 2012 conducted a study where they randomly collected 10,000 urine samples and tested for synthetic cannabinoids. That study reported a 2.5% positivity rate [13]. Synthetic cannabinoids are not tested for on routine clinical urine drug screens and so will often go undetected even in substance abuse treatment settings [14]. Part of the difficulty here is that there is a large and increasing number of distinct synthetic cannabinoids with diverse chemical structures being constantly synthesized making it difficult to develop assays for everyday clinical use. Compare this with cannabis which is the easiest substance of abuse to “catch” on urine drug screens [14]. This difficulty of detection along with governmental difficulty in efficiently identifying and legally controlling each new synthetic cannabinoid draws many people to them [13]. Synthetic cannabinoids are, sometimes dramatically so, associated with psychosis [15]. In regular cannabis use there is the low-affinity partial agonist THC acting on the CB1 receptor leading to many of the effects of cannabis use. In contrast, synthetic cannabinoids are full agonists with high affinity at the CB1 receptor [16]. Given this, it is not surprising that any deleterious effects from the former could be seen with greater severity and frequency with the latter. Indeed, much has been written about the harmful effects of synthetic cannabinoids including risk of psychosis [11,12,15,17,18,19,20,21]. 

The term psychosis in clinical settings refers to a plethora of abnormalities. Psychotic symptoms occur over a spectrum from acute to chronic and from mild to severe. Manifestations of psychosis are commonly broken down into “positive” and “negative” symptoms. Positive symptoms include delusions, hallucinations, disorganized thinking/speech/behavior, and disorganized or abnormal motor behavior. Negative symptoms include diminished emotional expression, avolition, alogia, anhedonia, and asociality [22] (pp 87–89). Positive symptoms are abnormal by their presence whereas negative symptoms represent abnormalities via absence of normal behaviors. Most of the reported associations between cannabis and psychosis, particularly for acute effects of cannabis use, focus on positive symptoms. However, there is some evidence of acute effects resembling negative symptoms as well [23,24]. 

Psychosis is merely a symptom whereas Schizophrenia is a chronic, lifelong illness, characterized by the presence of severe psychotic symptoms [22,25] (pp. 99–105). In addition to the chronicity required for a Schizophrenia diagnosis the concept of “first rank” psychotic symptoms has historically been used to help differentiate schizophrenia from other psychotic conditions [26]. First rank psychotic symptoms are relatively severe and are somewhat specific for Schizophrenia [27,28]. First rank psychotic symptoms include auditory hallucinations, delusional perceptions, experiences of thought interference, and passivity experiences [26,27]. Schizophrenia can lead to a devastating impairment in quality of life. Schizophrenia was responsible for 13.6 million disability-adjusted life years worldwide in 2010 [29]. Because schizophrenia confers extremely high morbidity and mortality it is understandable that so much attention has been paid to asking whether cannabis use increases one’s risk for developing it [30]. 

Cannabis is associated with a range of psychotic symptoms of widely variable severity. Cannabis is also associated with psychotic symptoms of widely variable timeframes. Cannabis-associated psychosis can be seen on the order of minutes, hours, days, or weeks in addition to the months and years timeframe seen in a schizophrenia diagnosis [6,31,32]. 

A holistic understanding of the link between cannabis and psychosis requires us to look at more than just schizophrenia. For the current review we will describe the association between cannabis and psychosis as it plays out in the context of three Diagnostic and Statistical Manual of Mental Disorders, fifth edition (DSM-5) diagnoses: Cannabis Intoxication, Cannabis-Induced Psychotic Disorder (CIPD), and Schizophrenia [22]. It is useful to use this lens because the DSM-5 criteria are very widely used and accepted. This gives us firmer footing to describe different “kinds” of cannabis-associated psychotic experiences than we would have otherwise. Delineating the plethora of cannabis/psychosis associations in the literature into these categories is merely meant as a useful way to conceptualize the associations and is not meant to strictly indicate the original works referenced in this review themselves were working with DSM-5 criteria. No single diagnostic framework is used consistently in the cannabis/psychosis literature with DSM-III, DSM-IV, DSM-5, ICD-8, ICD-9, and ICD-10 diagnoses all being used at different times as well as the use of a variety of clinical psychosis rating scales. 

## 2. Cannabis Intoxication

This is a diagnosis made when there is recent cannabis use, significant behavioral or psychological changes that developed during or shortly after cannabis use, and physical stigmata indicating the intoxication such as conjunctival injection or dry mouth [22] (p. 516). With respect to timing cannabis intoxication occurs within minutes for inhalational use but onset can take hours when cannabis is ingested. The symptoms typically last 3–4 hours but depending on dose and tolerance can persist up to 24 hours [33]. This is basically the standard cannabis “high” documented in the DSM-5 as a mental disorder in situations where it causes neuropsychiatric symptoms that are problematic.

Psychotic symptoms are not necessary for a cannabis intoxication diagnosis but can be part of the disorder with the caveat that insight must remain intact and the psychotic symptoms must not be sufficiently severe or persistent enough to warrant clinical attention for their own sake. If the symptoms are severe or persistent enough to warrant clinical attention for their own sake, then that would move us to a CIPD diagnosis. CIPD is discussed in the following section.

Most individuals meeting criteria for Cannabis Intoxication will not present for acute medical care, so looking at psychotic symptoms within this disorder gives us a sense of what psychotic symptoms can be associated with cannabis use in non-clinical populations. We can also note that the vast majority of worldwide cannabis users have at some point met criteria for a Cannabis Intoxication diagnosis (becoming intoxicated is to some degree the goal of any cannabis use) so the psychotic symptoms experienced therein have potential to effect a huge number of persons worldwide. Having described what Cannabis Intoxication is per the DMS-V we can look at the evidence associating cannabis and psychosis as might be seen within the parameters of this disorder.

A 2004 double-blind placebo-controlled experimental study by D’Souza et al that documented psychotic symptoms in healthy subjects after intravenous THC administration provides us with a straightforward and useful example [24]. By administering the Positive and Negative Syndrome Scale (PANSS) at different timepoints before and after intravenous THC administration, the transient or “intoxication” effects of THC with respect to psychotic symptoms were able to be followed. The PANSS is commonly used in research to monitor symptoms of psychosis [34]. The PANSS was administered 60 minutes prior to injection, 10 minutes after, 80 minutes after, and 200 minutes after. It was found that a modest mean increase in positive symptoms occurred and peaked 10 minutes after injection and returned to baseline by 200 minutes after injection. A transient increase in mean negative symptoms also was seen after injection and again symptoms returned to baseline by 200 minutes. Due to the study design using intravenous THC as opposed to inhaled or ingested THC the results seen here show quicker on/off effects than what would be experienced in the population at large where inhalation or ingestion are the common administration routes. The transient increases seen in this study in both positive and negative symptoms measured via PANSS peaked at approximate scores of 10. Putting these results in context the possible PANSS scores for either positive or negative symptom subscales are 7 to 49 and PANSS averages for schizophrenic persons have been reported at 18.2 for positive symptoms and 21.01 for negative symptoms [34]. So, we see that while increases in psychotic symptoms were seen in this study using healthy subjects the magnitude of symptoms was quite small and transient as mentioned above. Also, it is notable that a dose–response relationship was seen in this study with more psychotic symptoms occurring with 5 mg THC injection compared to 2.5 mg THC injection. This finding of an acute transient increase in psychotic symptoms after intravenous THC administration in healthy subjects was replicated by Morrison et al. in 2009 [35]. In human laboratory studies, concerning healthy individuals being administered THC at high doses, it has been approximated that 35–50% will experience psychotic symptoms [16].

The largest pool of evidence describing acute transient psychotic symptoms associated with cannabis use can be found in studies documenting general population cannabis users self-reported psychotic experiences during acute use. This data also gives us some sense of the proportion of cannabis users that experience psychotic effects acutely when using the drug in naturalistic settings. A 2003 review by Green et al. examined 12 studies that surveyed users’ subjective effects when using cannabis [36]. Three of the studies used open-ended questions to elicit subjective effects while nine studies used closed-ended questioning (checklists or questionnaires). All studies used had a sample size over 30. The open-ended studies found 2–14% of subjects reported hallucinations while 6–15% of subjects reported paranoia. The closed-ended questioning studies allowed for results to be combined when the surveys asked similar or identical questions about subjective effects of cannabis. Of subjects in closed-ended questioning studies, 19.8% reported hallucinations/visions (N = 3082), while 51.4% reported paranoia (N = 2708). It is interesting to note that close-ended questioning elicited more psychotic symptoms than open-ended questioning. Cannabis users were seen throughout these studies to endorse mostly beneficial effects when describing effects spontaneously and to endorse proportionally more harmful/bothersome effects when made to consider these via checklists and questionnaires. This is congruent with the cognitive biases typically associated with substance use disorders. It is interesting to see this even in this non-clinical population [37].

There is also evidence from a study by Sami et al. that former cannabis users were more likely to report having had psychotic experiences with cannabis than current cannabis users who were more likely to report pleasurable experiences [38]. Current users who indicated a future intention to quit were more likely to have had psychotic experiences with cannabis than current users who indicated no desire to quit. These findings (along with the differences Green et al reported with open vs closed questions) suggest a potential “in” for insight-driven interventions for helping people quit cannabis such as motivational interviewing.

As it is clear many users do not report psychotic effects from acute cannabis use it becomes important to ask what kind of person is at risk for these bothersome acute effects. Mason et al. looked at acute psychotic symptoms associated with cannabis use and stratified their cannabis users based on high or low pre-intoxication scores on the Schizotypal Personality Questionnaire (SPQ) [39]. SPQ was used as a proxy for baseline psychotic symptoms and can be taken to indicate risk or susceptibility to psychosis [40]. This study found greater acute transient effects on psychotic symptoms in individuals with higher SPQ scores at baseline. Acute effects were taken as the difference between Psychotomimetic States Inventory (PSI) scores 10–15 minutes after use and PSI scores 3–5 days later after at least 24 hours of cannabis abstinence. This result provides evidence that certain individuals, especially those experiencing some mild psychotic symptoms at baseline, are more prone to acute transient psychotic symptoms associated with cannabis use than others.

Having described some of the evidence for an acute association between cannabis and psychosis, as could be seen in a Cannabis Intoxication diagnosis, we will move on to describe Cannabis Intoxication’s more severe and persistent progeny, CIPD.

## 3. Cannabis-Induced Psychotic Disorder (CIPD)

Substance-Induced psychotic disorders are recognized by the DSM-5 and are placed in the category of Schizophrenia Spectrum and Other Psychotic Disorders. Substance-Induced psychotic disorders related to practically all substances of abuse can be described using this diagnosis [22] (pp 110–115).

A diagnosis of Cannabis-Induced Psychotic Disorder is given when one or both of hallucinations and delusions are present, the hallucinations and/or delusions developed during or soon after cannabis intoxication, the disturbance does not occur exclusively during the course of a delirium, and the disturbance causes clinically significant distress or impairment in social, occupational, or other important areas of functioning. Other criteria for the disorder are that cannabis should be thought to be capable of producing the disturbance seen and that the disturbance should not be able to be better explained by an independent psychotic disorder that is not cannabis-induced (such as pre-existing schizophrenia). The DSM-5 suggests that if symptoms last longer than one month a diagnosis other than CIPD should be considered [22] (p. 110).

Substance-induced psychotic disorders generally can occur in the context of recent intoxication or withdrawal from a substance (for example with alcohol) but in the case of cannabis only psychotic symptoms occurring in the context of recent intoxication are thought to appropriately lead to a CIPD diagnosis [22] (p. 114). 

Several things differentiate CIPD from Cannabis Intoxication. First and foremost is that in CIPD the hallucinations and/or delusions are the focus of the clinical presentation and are severe enough to warrant clinical attention/treatment as opposed to the psychotic symptoms that can be seen in Cannabis Intoxication which are more mild and self-limited and are not even required to make that diagnosis. A further distinction is that the hallucinations in CIPD are experienced without insight whereas in Cannabis Intoxication the hallucinations when present are experienced with insight intact and the DSM-5 linguistically downgrades these in places from frank hallucinations to “perceptual disturbances.” In addition to greater intensity/severity of symptoms CIPD can also have a much longer duration than Cannabis Intoxication. Cannabis Intoxication will necessarily resolve within 24 hours whereas CIPD can last for days and even weeks after cannabis exposure [6]. However, criteria for CIPD could also be met in a presentation only lasting on the order of hours if the symptoms are severe. 

The concept of a cannabis psychosis apart from simple intoxication has been recognized for literally hundreds of years—take the following example from 1779 describing a preparation of cannabis known as “Bangue”.

“Bangue is an intoxicating herb; in the use of which it is hard to say what pleasure can be found, it being very disagreeable to the taste and violent in its operation which produces a temporary madness, that in some, when designedly taken for that purpose, ends in running, what they call a muck, furiously killing every one they meet without distinction till themselves are knocked on the head like mad dogs [41] (p. 21).”

Another historical example of the recognition of CIPD consistent cannabis/psychosis association comes from French psychiatrist Dr Jacques-Joseph Moreau in 1845, describing the effects of hashish:

“acute psychotic reactions, generally lasting but a few hours, but occasionally as long as a week…illusions, hallucinations, delusions, depersonalization, confusion, restlessness and excitement [42].”

A 2016 study using emergency department data from Vallersnes et al gives us a registry data example of a cannabis/psychosis association consistent with CIPD [43]. This study searched a European database (Euro-DEN) that tracks Emergency Department (ED) visits for acute recreational drug toxicity. Over a one-year period across 16 centers they found 90 ED presentations where psychosis was a presenting complaint and acute cannabis use had occurred. In 31 of those presentations cannabis was the sole substance reported. This study excluded overdoses/self-harm presentations and allowed for substances documented to have been used acutely to be patient and observer reported. This second distinction is particularly important when trying to assess association between acute cannabis use and CIPD-consistent psychosis since lab-testing for cannabis can be positive long after acute ingestion.

Unfortunately, most of the literature with respect to documented cases consistent with CIPD diagnoses is limited to case reports and case series and many of the oft-cited examples are from decades ago. In general we can say that these case reports and series describe acute cannabis use, psychotic symptoms severe enough to bring the individual to medical attention, symptoms occurring immediately after cannabis use, and return to baseline several hours to weeks after ingestion [44,45,46,47,48,49,50,51]. The one large study by number of subjects (N = 36,000) looked at American soldiers in Germany and documented some cases of “toxic psychosis” and “schizophrenic reactions” but did not control for use of other drugs and alcohol [51]. One of these studies had follow-up enough to document that the individuals who later relapsed with respect to cannabis use uniformly had recurrence of psychotic symptoms [49].

The return to baseline functioning as documented in these case reports is crucial in order to maintain the concept of CIPD. The difficulty in confidently diagnosing CIPD has been widely noted, as confirming absence of prior prodromal or psychotic symptoms and then also confirming return to baseline is quite difficult [31,52,53]. 

The “fuzziness” of the diagnosis and the dissimilar situations where it applies leads to confusion. CIPD criteria are met both in cases of extreme intoxication where psychotic symptoms overwhelm the clinical picture but may be very short lived, and also in situations that—apart from knowledge of recent cannabis exposure—could appear identical to a first break psychosis as is seen in schizophrenia (i.e. requiring extended hospitalization and antipsychotic medication).

Despite these problems CIPD remains an important diagnostic construct that should not be ignored. CIPD allows us to conceptualize that there is a middle ground in the cannabis/psychosis association between simple intoxication and long-term psychosis. In the following section we will describe evidence linking CIPD to later schizophrenia diagnoses, thus completing a diagnostic chain from Cannabis Intoxication to CIPD to schizophrenia.

## 4. Schizophrenia

Schizophrenia is the prototypical psychotic disorder and is characterized by its chronicity and severity. Historically a schizophrenia diagnosis has required the presence of so-called first rank symptoms indicating severe psychosis [26,28]. Schizophrenia is quite common with a global prevalence of approximately 0.7% [54,55]. 

DSM-5 diagnostic criteria for schizophrenia are quite detailed [22] (p. 99) so let us paraphrase here by saying schizophrenia is diagnosed when there are multiple psychotic symptoms present coupled with a decreased level of work and/or social functioning and the total duration of the disturbance is greater than 6 months. Two or more active-phase psychotic symptoms including delusions, hallucinations, disorganized speech, grossly disorganized or catatonic behavior, and negative symptoms must be present at least a month if untreated. One of the active-phase symptoms must be either delusions, hallucinations, or disorganized speech [22] (p.99). The remainder of the six-month period required to make the diagnosis can include prodromal or residual/attenuated symptoms only. The DSM-5 does not make explicit reference to the historical concept of first rank symptoms however these symptoms are part of a DSM-5 Schizophrenia diagnosis in most cases given that one of delusions, hallucinations, or disorganized speech is required by the DSM-5 and delusions and hallucinations are first rank symptoms [25,27,28].

The association between cannabis and schizophrenia has been a heavily researched and debated topic in the literature and rightly so. Schizophrenia has a huge morbidity/mortality burden and if cannabis is a cause or a component cause it would be a highly modifiable risk factor for this devastating disease [29].

Interest in the cannabis/schizophrenia association was sparked by a study using Swedish military conscription data led by Andreassson [56]. This data represents >97% of the age 18–20 male population of Sweden from 1969. Data on substance use including cannabis was collected at time of conscription and schizophrenia outcomes over the next 15 years were collected and matched with the subjects’ initial reports of cannabis use. The study documented an increased risk for schizophrenia in those who had ever used cannabis prior to their conscription and documented a dose–response relationship with respect to number of lifetime uses of cannabis and schizophrenia risk. Zammit et al conducted a 27-year follow-up of the same cohort and re-analyzed the data [57]. The number of subjects analyzed was 50,053. The Zammit et al. study reported an odds ratio for schizophrenia of 2.2 for ever using cannabis and an odds ratio of 6.7 for those who had used cannabis more than 50 times. The effect remained after adjusting for some potential confounders including psychiatric diagnoses at conscription, IQ score, personality variables concerned with interpersonal relationships, place of upbringing, paternal age, and cigarette smoking. The adjusted odds ratio for ever using cannabis was 1.5 and the adjusted odds ratio for >50 cannabis uses lifetime use was 3.1. The association between cannabis use and a chronic psychotic disorder (either schizophrenia or schizophreniform disorder) in longitudinal studies has been replicated multiple times [58,59].

A 2016 meta-analysis of 10 studies (66,816 total individuals across the studies) looking at the association between degree of cannabis use and subsequent psychotic symptoms found an overall OR of 3.90 for the heavy users compared to never users [60]. The studies used in this meta-analysis had at least three groups of cannabis use: never; one or more intermediate levels of use; and a “heavy” level either by duration of cannabis use, frequency of cannabis use, or total number of times cannabis had been used lifetime. This meta-analysis included outcomes other than just chronic psychotic disorders but is very useful as evidence that the dose–response relationship is robust.

It has been reported in a case control study that amongst patients with psychotic disorders those who used cannabis daily, those who used higher potency cannabis, and those who started at a younger age tended to experience the onset of psychotic symptoms earlier than those psychotic disorder patients who did not use cannabis in the same high-risk ways [61]. This can be taken as more evidence of a dose–response relationship. 

There is also a well-established association between CIPD and later schizophrenia. A study using Danish registry data from 1994–2014 examined the proportion of patients given substance-induced psychotic disorder diagnoses that would go on to later be given schizophrenia or bipolar diagnoses [62]. These were patients that did not have schizophrenia or bipolar disorder diagnoses before the incident substance-induced psychotic episode. In this registry study it was reported that 41.2% of patients with cannabis-induced psychotic disorder eventually converted to schizophrenia. A total of 47.4% of patients with cannabis-induced psychotic disorder eventually converted to either schizophrenia or bipolar disorder.

It is intuitive that having a substance-induced psychotic episode, whatever the offending substance, could be a substantial risk factor for future psychiatric morbidity. That said, in the same Danish registry study, cannabis had the highest rate of conversion from substance-induced psychosis to schizophrenia or bipolar disorder out of all substances investigated. Compare that 47.4% rate for cannabis to 32.3% for amphetamines, 20.2% for cocaine, 27.8% for hallucinogens, and 35.0% for mixed/other substances. Fifty percent of the cannabis-induced psychosis patients that converted to schizophrenia did so within 3.1 years of the incident psychotic episode while the remaining 50% that ended up converting to schizophrenia did so over many years. This delayed conversion after the incident CIPD episode can be looked at as evidence for the CIPD episode being its own entity as opposed to a mis-diagnosed first episode of schizophrenia.

Other registry studies have also found persons with CIPD-consistent presentations to have a high rate of conversion to schizophrenia. A Swedish registry study for substance-induced psychosis converting to schizophrenia found cannabis to have the highest conversion rate of all substances at 18% [63]. A Finnish study of 18,478 inpatient case calculated that 46% of CIPD cases converted to schizophrenia and this was the highest percentage for any substance [64]. A study using Scottish data found 21.4% of people with cannabis-induced psychotic disorder eventually converted to schizophrenia [65]. In that study cannabis had a lower conversion rate than cocaine and solvent-induced psychoses however the N’s for cocaine and solvent-induced disorders were very small (24 and 14 respectively compared to 276 for cannabis). In the Scottish study “multiple substance” or “other” substance-induced psychoses showed a conversion rate of 21.5%. Compared to the Danish, Swedish, and Finnish studies the Scottish data found cannabis-induced psychotic disorder conversion to schizophrenia to be more similar to the rates for other substances.

It is important to note that most of these registry studies are looking at cannabis use in relatively young people and subsequent schizophrenia or other psychotic outcomes. Age of onset of cannabis use appears to heavily influence the cannabis/schizophrenia association [9]. One potential explanation for this is that cannabis use has stronger effects on developing brains and that is what leads to a stronger association with future psychoses.

Genetic risk is an important part of the cannabis/schizophrenia association as well. We should expect this as schizophrenia has often been estimated as having approximately 80% heritability [66]. A study from Gage et al used single nucleotide polymorphisms (SNPs) associated with cannabis initiation and SNPs associated with schizophrenia to calculate a small causal effect (OR = 1.04) of cannabis initiation on subsequent schizophrenia [67]. This study also illustrated the complex and seemingly bidirectional nature of the cannabis/schizophrenia association, calculating a stronger causal effect of schizophrenia on cannabis initiation (OR = 1.10). Another genetic study using SNPs found a similar result with OR = 1.1 for cannabis being causally implicated in schizophrenia and OR = 1.16 for the reverse [68]. 

A specific example of genetic involvement in the cannabis/schizophrenia association can be seen in the COMT gene. The COMT gene codes for the enzyme catechol-O-methyltransferase which is important in the breakdown of dopamine, particularly in the prefrontal cortex [69]. The Val158Met SNP is a Methionine to Valine substitution that causes an alteration of enzyme activity. Val/Val homozygotes have the highest enzymatic activity, Val/Met heterozygotes have intermediate activity, and Met/Met homozygotes have the lowest activity. This results in Val/Val homozygotes depleting dopamine the fastest and Met/Met homozygotes the slowest. Dysregulation of dopamine has long been considered a crucial part of the pathophysiology of schizophrenia and a great deal of research has been done to investigate the link between COMT polymorphisms and schizophrenia, particularly with respect to negative symptoms and cognition [69,70]. These studies have shown a variety of interesting results including some studies demonstrating significant interactions between cannabis use and COMT genotype and development of schizophrenia [17,71,72,73,74,75,76,77]. In 2005 Caspi et al reported data on 803 individuals born in Dunedin, New Zealand (known as the Dunedin cohort) [76]. These individuals were followed up periodically from ages 3 to 26. This study found that 13% of adolescent cannabis users with the Val/Val genotype met criteria for schizophreniform disorder at age 26 while only 1.4% of non-cannabis-using adolescents with the same Val/Val genotype met criteria for the disorder. Schizophreniform disorder has the same DSM-5 criteria as Schizophrenia, but this diagnosis is given when the symptoms are only known to have lasted from 1–6 months. The odds ratios calculated for adolescent cannabis use and subsequent schizophreniform diagnosis for the three genotypes were 10.9 for Val/Val, 2.5 for Val/Met, and 1.1 for Met/Met. Genotype by itself without the covariant of cannabis use was not found to be significantly associated with subsequent schizophreniform diagnosis. The impressive results from this oft-cited study demonstrate very well the concept that there appears to be an important gene–environment interaction to be considered when assessing the cannabis/schizophrenia link. However, attempts to replicate this study have been mixed with both positive and negative results [78,79,80,81,82,83,84,85]. Subsequent positive results have been seen in studies considering the combined interaction between COMT genotype, cannabis use, and history of childhood abuse and subsequent schizophrenia [81,85]. 

Another example of the COMT gene’s role in the cannabis/schizophrenia association is seen in a study from Pelayo-Terán et al. published in 2009 [77]. This study looked at 169 patients in Spain with first-episode psychosis and examined the interaction between COMT genotype and cannabis use and age of onset of psychotic symptoms and duration of untreated psychosis prior to treatment presentation. This study found that low enzymatic activity Met/Met patients who were not cannabis users tended to have a later age of onset of psychosis and a longer period of untreated psychosis compared to Val/Val or Val/Met cannabis non-users. Longer period of untreated psychosis can be considered a proxy for more mild symptoms or primarily negative symptoms as it is expected that severe positive symptoms will be what brings patients to acute medical attention. Based on this data the Met/Met genotype can be considered something of a protective factor against severe/early disease. The most salient finding of the study was that cannabis users with the Met/Met genotype did not have the delayed onset or longer period of untreated psychosis seen in Met/Met non-users. This suggests that cannabis use changes the natural course of psychotic symptoms typically seen with the Met/Met genotype. This study also found that cannabis users of any COMT genotype experienced an earlier onset of symptoms compared to non-users.

In addition to discussing the cannabis/psychosis association with respect to the onset of schizophrenia we can discuss the impact of cannabis use on people who already have schizophrenia. D’Souza et al conducted a double-blind placebo-controlled study where intravenous THC was administered to Schizophrenia patients already in treatment for schizophrenia and maintained on stable antipsychotic dosages [86]. The study design was the same as the study on healthy subjects from the same author described above in the Cannabis Intoxication section of this review [24]. Similar results were found in the study of healthy individuals with transient increases in positive and negative symptoms seen via PANSS (although as expected the baseline scores were higher in the schizophrenic population). It is important to point out that these exacerbations in psychotic symptoms with cannabis administration were seen despite the schizophrenic patients being on dopamine-blocking antipsychotic drugs. A higher percentage of schizophrenia patients experienced transient symptom exacerbations compared to the study with healthy persons [6].

Further, schizophrenia patients with a cannabis use history compared to schizophrenic patients without a cannabis use history have been documented to have longer and more frequent psychiatric hospital stays which would seem to indicate a higher symptom burden [87].

## 5. Discussion

The association between cannabis use and psychosis is important for all stakeholders to understand. Cannabis users, potential future users, existing schizophrenia patients, families of at-risk persons, researchers, clinicians, and policy-makers all need to be aware of the multi-modal and complex relationship cannabis use has to a variety of psychotic outcomes in order for harm to be reduced and appropriate informed consent be achieved. A measured appreciation of the nuances will be necessary for rational decision-making going forward. 

Rational decision-making regarding this issue is particularly salient at the current time as legalization (especially in the United States) expands, public perception trends towards considering cannabis benign or medicinal, and the very nature of the substance being used is functionally changing vis-à-vis increased potency [4,88,89,90]. 

The DSM-5 dominates mental health nosology and so considering the various associations between cannabis and psychosis in terms of the DSM-5 allows for a better understanding of this complex issue. Associations between cannabis and psychosis span the full range from mild to severe and hyper-acute to lifelong. Severity and chronicity are usually positively correlated when it comes to cannabis-associated psychosis but not always. Psychosis can be seen even with the mildest of the cannabis-related disorders in the DSM-5, Cannabis Intoxication. When psychotic symptoms associated with cannabis exceed the threshold of attracting medical attention and/or persist beyond 24 hours that moves us to a CIPD diagnosis. Awareness of CIPD is key to conceptualizing the full continuum of cannabis and psychosis associations. CIPD can lead to significant distress and impairment on its own and is heavily associated with future Schizophrenia [62,63,64,65]. Diagnosis of CIPD is seemingly simple but actually quite complex, as to appropriately diagnose a case of CIPD absence of symptoms prior to cannabis exposure and return to baseline both need to be known. 

The temporality and specificity of cannabis intoxication and CIPD (exposure and then immediate symptoms) allow us, with reasonable confidence, to say cannabis use “causes” these conditions. The etiology of schizophrenia on the other hand is very complex and no one factor can be said to cause schizophrenia. Rather, a multitude of potential component causes influence the likelihood of schizophrenia via their presence or absence. Cannabis use appears to be a component cause of schizophrenia but is neither necessary nor sufficient.

Looking at Hill’s criteria for assessing causation and comparing them to the evidence for the cannabis/schizophrenia association we see we have consistency, temporality, biological gradient, plausibility, coherence, experiment, and analogy. Weaknesses for cannabis/schizophrenia causality include the strength of the association often being relatively small (especially when correcting for confounders) and confidence with respect to specificity being very difficult to achieve [91]. For Cannabis Intoxication and CIPD all of Hill’s criteria can be accounted for.

In closing we would point out one glaring weakness in the literature regarding biological gradient. Several studies have documented some form of dose–response but there is little-to-no evidence available on THC/CBD ratios or gross CBD amounts in the cannabis used by those who do or do not then go on to have psychotic outcomes. This is important data to collect as there is evidence that CBD tends to ameliorate psychotic symptoms associated with THC [92].
